# Determination of nine prostaglandins in the arachidonic acid metabolic pathway with UHPLC-QQQ-MS/MS and application to *in vitro* and *in vivo* inflammation models

**DOI:** 10.3389/fphar.2025.1595059

**Published:** 2025-05-30

**Authors:** Yufeng Huang, Mengxian Wang, Ziqi Yang, Xiaotong Wang, Xinxin Wang, Fan He

**Affiliations:** ^1^ Guangdong Provincial Hospital of Chinese Medicine, The Second Affiliated Hospital of Guangzhou University of Chinese Medicine, Guangzhou, Guangdong, China; ^2^ College of Chinese Medicine, Guangzhou University of Chinese Medicine, Guangzhou, Guangdong, China; ^3^ Affiliated Hospital of Liaoning University of Traditional Chinese Medicine, Shenyang, Liaoning, China

**Keywords:** arachidonic acid, prostaglandins, PGE_2_, PGD2, UHPLC-QQQ-MS/MS, inflammation

## Abstract

**Background:**

Prostaglandins play a vital role as crucial metabolites and inflammatory indicators within the arachidonic acid (AA) metabolic pathway. Conventional assays typically focus on a single inflammatory indicator, while multi-index detection entails a large number of samples.

**Methods:**

In this study, an ultra-high-performance liquid chromatography coupled with triple quadrupole mass spectrometry (UHPLC-QQQ-MS/MS) method was newly developed for simultaneous quantitative analysis of nine AA metabolites, including prostaglandin F2β (PGF_2β_), prostaglandin E2 (PGE_2_), prostaglandin E1 (PGE_1_), prostaglandin D1 (PGD_1_), prostaglandin D2 (PGD_2_), prostaglandin A2 (PGA_2_), prostaglandin J2 (PGJ_2_), prostaglandin B2 (PGB_2_), and prostaglandin A1 (PGA_1_), in the supernatant of LPS-induced RAW264.7 cells and the serum samples of adjuvant-induced arthritis (AIA) rats.

**Results:**

The newly established UHPLC-QQQ-MS/MS method successfully and rapidly quantified the contents of the nine prostaglandins simultaneously. The methodology was validated. The levels of PGE_2_, PGD_1_, PGD_2_, PGA_2_, and PGJ_2_ in the LPS-induced RAW264.7 cells group were higher than those in blank group. At the same time, the levels of these PGs decreased significantly (*p* < 0.01 vs. LPS-induced group) after the positive drug (dexamethasone) intervention. On the 14th day of AIA modeling, the paw volume of the AIA rats was significantly enlarged (*p* < 0.01 vs. blank group), and the serum samples from the AIA group showed significantly increased levels of PGE_2_, PGD_2_, and PGA_2_ (*p* < 0.01 vs. blank group), suggesting the emergence of arthritis. The levels of other prostaglandins were below the limit of quantification.

**Conclusion:**

The method established in this study for determining nine prostaglandins in the AA metabolic pathway with UHPLC-QQQ-MS/MS embodied the advantages of requiring a low amount of sample, a simple pretreatment process, and the rapid and efficient simultaneous quantification of multiple inflammatory factors. It provided a novel assay method for the pharmacological study of the AA metabolic pathway.

## 1 Introduction

**FIGURE 1:**
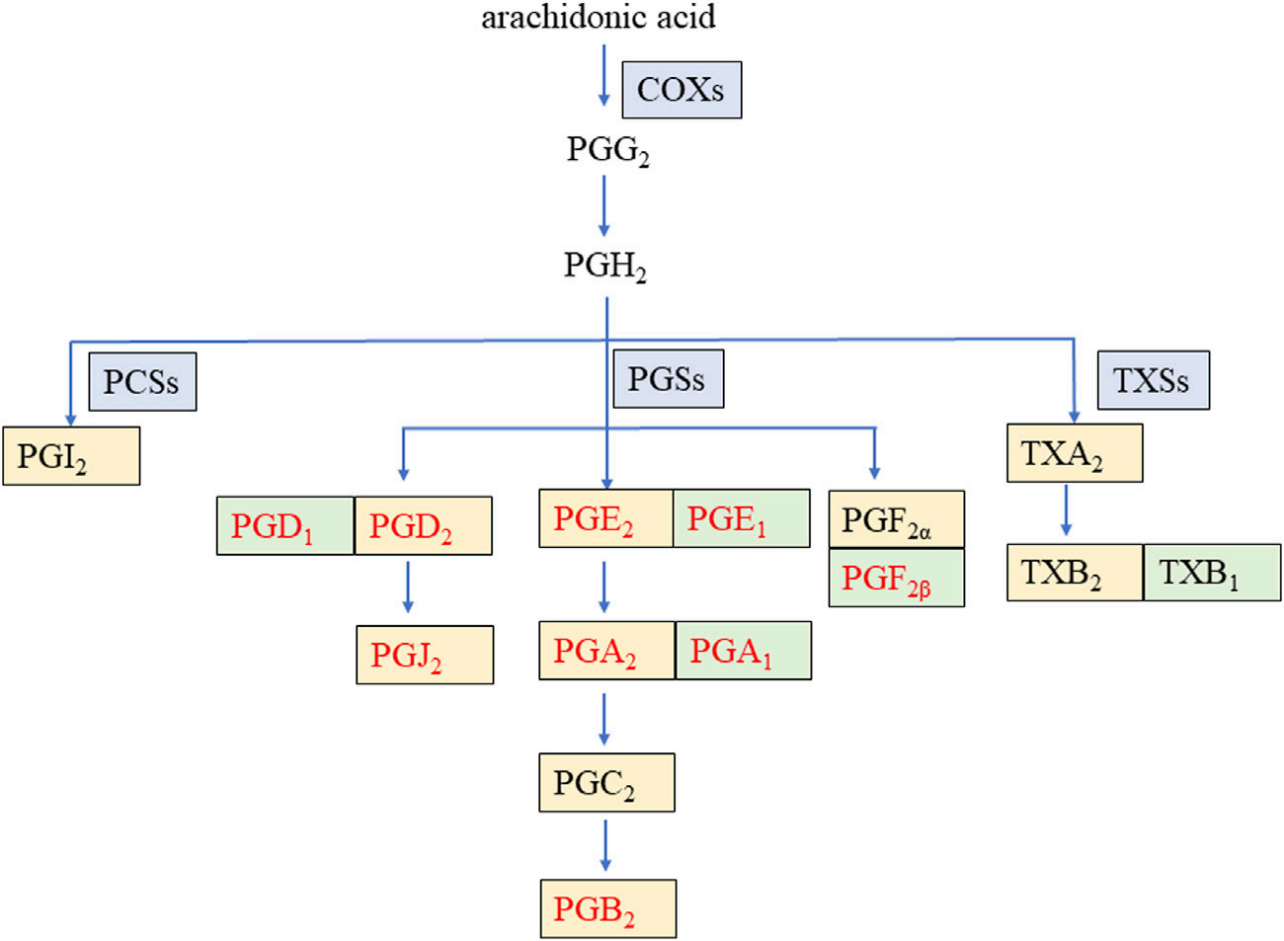
Arachidonic acid metabolic pathway of some prostaglandins during inflammation. COXs: cyclooxygenases, PCSs: prostacyclin synthetases, PGSs: prostaglandin synthetases, TXSs: thromboxane synthases, PG: prostaglandin, TX: thromboxane. The enzyme is on a blue background. The pro-inflammatory factor is on a yellow background. The anti-inflammatory factor is on a green background. The nine PGs detected in this study are in red font.

Prostaglandins (PGs) represent a class of lipid compounds within the arachidonic acid (AA) metabolic pathway, possessing diverse bioactivities. They are intricately involved in crucial processes such as inflammation, pain transmission, and immune regulation, and they play an indispensable role in the pathophysiological mechanisms of numerous diseases, including arthritis, cardiovascular disease, and tumors (Zhang et al., 2020). As depicted in Figure 1, AA, under the catalytic action of cyclooxygenases (COXs), is converted into the intermediate metabolites prostaglandin G2 (PGG_2_) and prostaglandin H2 (PGH_2_). Subsequently, these intermediates are rapidly metabolized by distinct downstream prostaglandin synthases, giving rise to a plethora of pro-inflammatory active PGs, such as prostaglandin E2 (PGE_2_), prostaglandin D2 (PGD_2_), prostaglandin F2α (PGF_2α_), and prostaglandin I2 (PGI_2_) (Wang et al., 2021). Conversely, certain PGs, namely, prostaglandin F2β (PGF_2β_), prostaglandin E1 (PGE_1_), prostaglandin D1 (PGD_1_), and prostaglandin A1 (PGA_1_), can interact with and modulate the activity of specific proteins, thereby exerting an anti-inflammatory effect (Code et al., 2021; Amagai et al., 2015).

Currently, in pharmacological research, the detection of pro- and anti-inflammatory factors, such as PGs in the AA metabolic pathway, is predominantly performed by enzyme-linked immunosorbent assay (ELISA), real-time polymerase chain reaction (RT-PCR), and Western blot (WB). Nevertheless, these assays are capable of only detecting one index at a time, with low detection efficiency, high detection cost, and high technical requirements for operators (Yu et al., 2024). The liquid phase combined mass spectrometry (LC-MS) technique serves as a primary tool that is frequently employed in metabolomics to detect endogenous compounds, which has the advantages of high sensitivity, short analysis time, and assay automation (Yang et al., 2023; Wang Y. et al., 2023). It has been reported that the LC-MS methods quantitatively determined the AA and PGE_2_ in human plasma (Gachet et al., 2015) and PGE_2_ in acute spinal cord injury samples of rats (Pang et al., 2022), respectively. However, the existing reported LC-MS methods detect only 1–2 metabolites in the AA pathway at a time, which neither comprehensively reflects the changes of most PG metabolites in the metabolic pathway nor utilizes the advantage of LC-MS that can simultaneously determine multiple target components.

Therefore, in this study, a novel, rapid, and sensitive ultra-high-performance liquid chromatography coupled with triple quadrupole mass spectrometry (UHPLC-**QQQ**-MS/MS) method was developed. This method enabled the simultaneous and successful detection of the contents of nine PGs in samples. In addition, the newly established LC-MS method was applied to preliminarily explore the variation patterns of these nine PGs in two models: the lipopolysaccharide (LPS)-induced RAW264.7 cell model, which is a classical *in vitro* inflammation model, and the rat adjuvant-induced arthritis (AIA) model, a representative *in vivo* inflammation model. The developed method offers several advantages, including a low sample requirement, simple sample pretreatment procedures, and the ability to rapidly and efficiently perform simultaneous quantification of multiple PGs. Thus, it provides a novel analytical approach for efficiently investigating the alterations in the metabolite content within the AA metabolic pathway during the inflammation process.

## 2 Materials and methods

### 2.1 Chemicals and reagents

Reference compounds, each with a purity of ≥98%, including PGF_2β_, PGE_2_, PGE_1_, PGD_1_, PGD_2_, PGA_2_, PGJ_2_, PGB_2_, PGA_1_, and PGA_2_-D4 (which served as an internal standard substance, IS), were purchased from Cayman Chemical Company (United States). HPLC-graded formic acid, acetonitrile, and methanol were purchased from Macklin (China), AQA (United States), and Fisher Scientific (United States), respectively. Ultrapure water was generated by a water purification system (RODI-220A1, RSJ, China). Dulbecco’s Modified Eagle Medium (DMEM), fetal bovine serum, and phosphate-buffered saline (PBS) were all purchased from Gibco (United States). LPS was purchased from Sigma-Aldrich (United States). Complete Freund’s adjuvant (CFA) and inactivated *mycobacterium tuberculosis* (Mtb) were acquired from BD (United States). Mineral oil was bought from Sigma-Aldrich (United States).

### 2.2 Experimental instrumentation and LC-MS conditions

First, an ultra-high-performance liquid chromatography Agilent 1290 system integrated with Agilent 6495C triple quadrupole mass spectrometer (UHPLC-QQQ-MS/MS, Agilent Technologies, Santa Clara, United States) was employed for mass spectrometry optimization of the nine reference compounds and IS. Subsequently, quantitative detection was carried out.

The analytes in the mixed reference solution and test sample solutions were chromatographically retained on a Waters ACQUITY UPLC HSS T3 column (1.8 μm, 2.1 mm × 100 mm, Waters, Milford, United States). The mobile phases consisted of 0.1% formic acid–water (A) and acetonitrile (B), with a gradient elution program as follows: from 0 to 3 min, the proportion of B was maintained at 40%, and from 3.01 to 8 min, the proportion of B was increased from 40% to 80%. An aliquot of 5 μL of the sample was injected for analysis, and the flow rate was set at 0.30 mL/min.

In the negative ion mode, the multi-reaction monitor (MRM) mode, in combination with an electrospray ionization (ESI) source, was utilized to detect the nine metabolites. The other parameters were as follows: the flow rate of dry gas (N_2_) was 11.0 L/min, the dry gas temperature was 300°C, the nebulizer was 15 psig, and the capillary voltage was 4,000 V.

### 2.3 Cells and animals

#### 2.3.1 Establishment of LPS-induced RAW264.7 cells

RAW264.7 cells were purchased from Starfish Biologicals (China). Cell modeling and grouping were carried out according to previous reports in our laboratory (Guo et al., 2023). Briefly, cells in the logarithmic growth phase were laid in 12-well plates, 1.5 × 10^5^/well, and the blank group, model group (100 ng/mL LPS), and positive drug group (0.5 μM dexamethasone, DEX) were set up, with three replicate wells in each group. After the cells were adhered to the wall, the positive drug group was pre-administered with LPS for 1 h before stimulation. The cells were incubated at 37°C with a 5% CO_2_ incubator, and the cell supernatant of each group was collected for the next experiment after 24 h of incubation. PBS was added to each group of cells for microphotography to determine whether the modeling was successful.

#### 2.3.2 Establishment of the AIA model in rats

A total of 12 male Sprague-Dawley (SD) rats (180 g–220 g) were purchased from Guangdong Province Viton Lihua Laboratory Animal Technology Co., Ltd. The rats were housed in the specific pathogen-free (SPF) Animal Laboratory of Guangdong Provincial Hospital of Chinese Medicine at an ambient temperature of 23°C–25°C, relative humidity of 45%–70%, and 12 h/12 h day/night alternation, with free access to food and water. The Ethics Committee of the Guangdong Provincial Hospital of Chinese Medicine evaluated the animal experiments with ethics No. 2023124.

Six healthy male SD rats were randomly selected for AIA modeling. Rats were immunized by subcutaneous injection at the base of the tail with CFA mixed with Mtb according to the method described by our lab previously (Pan et al., 2019). The six remaining rats were used as a blank group. The blank group was injected with an equal dose of sterile PBS. On the seventh day after the initial immunization, the above procedure was repeated by injecting 0.15 mL of CFA into the root of the rats’ tails. On the 14th day, the modeling of the rat AIA model was completed.

A digital water plethysmometer (LE7500, Panlab, Spain) was used to measure the swelling volume of the hind paws before and after modeling in rats to evaluate whether the modeling was successful.

### 2.4 Sample pretreatment and methodology

#### 2.4.1 Configuration of the standard solutions

An appropriate amount of PGF_2β_, PGE_2_, PGE_1_, PGD_1_, PGD_2_, PGA_1_, PGA_2_, PGJ_2_, and PGB_2_ was precisely weighed as reference compounds, respectively, and a single reference stock solution with methanol was prepared. Then, a mixed reference solution was formed with the appropriate amount of each single reference stock solution. The content of PGF_2β_, PGE_2_, PGE_1_, PGD_1_, PGD_2_, PGA_1_, PGA_2_, PGJ_2_, and PGB_2_ in the mixed reference solution was 1.07, 1.11, 0.550, 0.400, 1.12, 0.369, 0.100, 0.150, and 0.200 μg/mL, respectively.

#### 2.4.2 Configuration of the internal standard solution

An appropriate amount of PGA_2_-D4 was taken, methanol was added to form a reserve solution of 0.1 mg/mL, and it was stored at 4°C. Before use, methanol was diluted to 0.1 μg/mL as the internal standard working solution.

#### 2.4.3 Pretreatment of the cell supernatant samples

First, 200 μL of the cell supernatant was pipetted into a 1.5 mL EP tube. Then, 50 μL of PGA_2_-D4 and 800 μL of acetonitrile were added to the cell supernatant, and the mixture was thoroughly vortex-mixed to ensure homogeneity. Subsequently, the resulting mixture was centrifuged at 4°C at a rotational speed of 14,000 rpm for 10 min. After centrifugation, the supernatant was carefully collected. The collected supernatant was then dried using a nitrogen blower. Following the drying step, the residue was re-dissolved in 100 μL of methanol. Finally, this methanol-based solution was centrifuged again at 4°C at 14,000 rpm for 10 min, and the supernatant obtained was taken for LC-MS determination.

#### 2.4.4 Pretreatment of rat serum samples

On the second day following successful model establishment, the rat blood sample was taken from the abdominal aorta after anesthesia into a 1.5 mL EP tube. The blood was centrifuged at 3,500 rpm for 10 min at 4°C as soon as possible. The supernatant obtained from the centrifugation was carefully collected and reserved for the subsequent experiment. A 200-μL aliquot of the rat serum was then pipetted, and the subsequent preparation process was identical to that described in sections 4.3 and 2.4.3.

#### 2.4.5 Method validation

The LC-MS method validation of the cell supernatant and rat serum samples was conducted according to the bioanalytical method validation guidelines from the US Food and Drug Administration (US Food and Drug Administration, 2018). Method validation was well-studied, including specificity, linearity, precision, recovery, and stability.

### 2.5 Data analysis

LC-MS data analysis was carried out using MassHunter Workstation software Quantitative Analysis (Agilent Technologies, Santa Clara, United States). The sample concentrations were evaluated via linear regression analysis. Images and data were counted and processed using GraphPad Prism 10.0 software (GraphPad Software, Boston, United States). *P* < 0.05 was considered a statistically significant difference. All data are expressed as the mean ± standard deviation.

## 3 Results

### 3.1 LPS-induced RAW264.7 cells model

**FIGURE 2:**
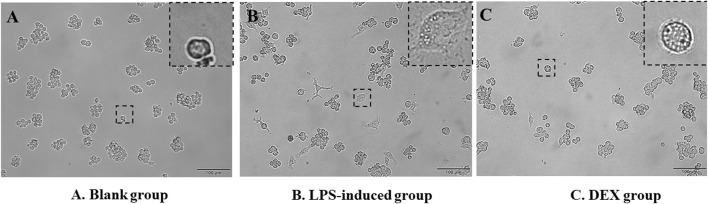
Morphology of RAW264.7 cells’ appearance. **(A)** RAW264.7 cells as a blank group. **(B)** LPS-induced RAW264.7 cells as a LPS-induced group. **(C)** Dexamethasone-pretreated LPS-induced RAW264.7 cells as a DEX group.

LPS-induced RAW cells successfully constructed an *in vitro* inflammatory model, as judged by the morphology of cell appearance. Microscopic observation showed that RAW264.7 cells were round in the normal state, with smooth cell edges without pseudopods and no vacuoles in the cytoplasm (Figure 2A). After 24 h of LPS stimulation, the surface area of the cells became larger, pseudopods were protruded, and intracytoplasmic vacuoles increased (Figure 2B). The administration of DEX as a positive drug protected the stimulation of the cells (Figure 2C).

### 3.2 AIA rat model

**FIGURE 3:**
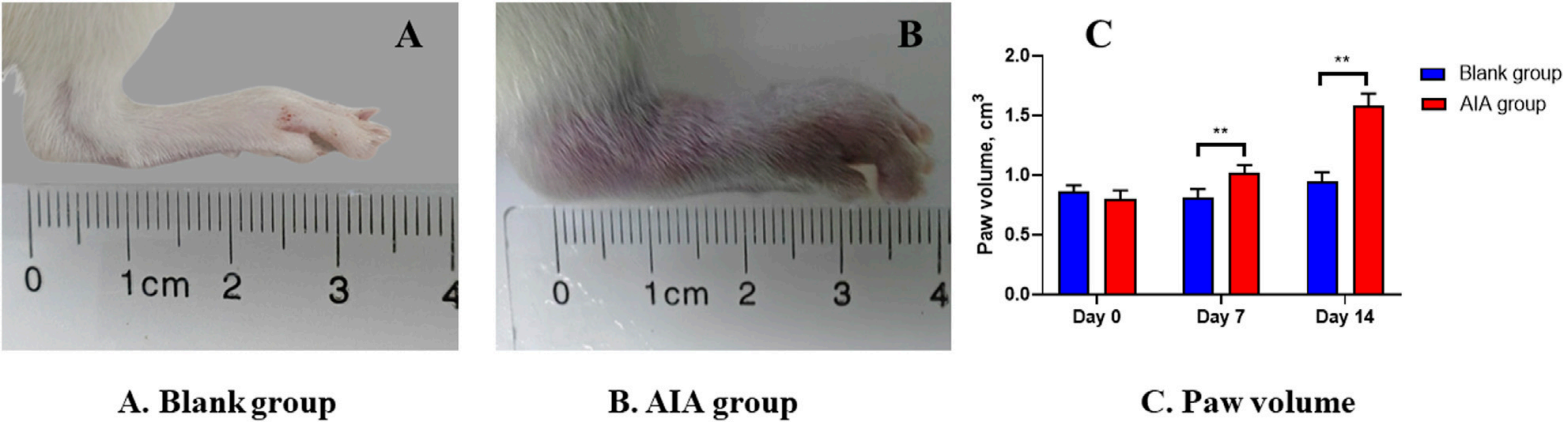
Changes of apparent observation and paw volume of AIA rat modeling. **(A)** Blank group: the healthy rat did not show hind paw swelling. **(B)** AIA group: the hind paw of the model rat was obviously red and swollen. **(C)** Paw volume: on the seventh and 14th day of modeling, the volume of the hind paw in the AIA group was significantly enlarged. Paired t-tests were used for statistical analyses. **P < 0.01, vs. blank group, *n* = 6.

The results of apparent observation showed that the AIA rat modeling was successful. Since day 7 of modeling, redness and swelling began to appear in the foot extremities of rats in the model group. With the progression of the disease, the redness and swelling gradually expanded after the second booster immunization. The hind paws of the model group (Figure 3B) exhibited apparent redness and swelling, consisting of the characteristics of active arthritis compared with the blank group (Figure 3A). The paw volume of rats in each group was measured by drainage method on day 0, day 7, and day 14. Figure 3C shows that the paw volume in the AIA group was significantly higher than that in the blank group on day 7 and day 14 (*p* < 0.01).

### 3.3 Method validation

#### 3.3.1 Method specificity

Under the selected chromatographic conditions in Section 2.2 and Table 1, the target metabolites and IS contained in the biological samples can be entirely separated, with symmetrical peak shapes and no interference from endogenous substances and sample matrix (Supplementary Figures S1, S2).

**TABLE 1:** Ion optimization conditions of the reference compounds.

References compound	Parent ion (m/z)	Daughter ion (m/z)	Fragmentor voltage (V)	Collision energy (eV)	ESI mode
PGF_2β_	353.2	309.1	166	20	Negative
PGE_2_	351.2	271.2	166	16	Negative
PGE_1_	353.2	317.1	166	16	Negative
PGD_1_	353.2	317.1	166	12	Negative
PGD_2_	351.2	271.1	166	16	Negative
PGA_2_	333.2	271.1	166	16	Negative
PGJ_2_	333.2	271.1	166	16	Negative
PGB_2_	333.2	235	166	20	Negative
PGA_1_	335.2	273.1	166	20	Negative
PGA_2_-D4	337.2	239	166	20	Negative

#### 3.3.2 Linearity

The linear calibration curves were obtained in the given concentration range of each PGs in samples, respectively. The standard curves were fitted to a first-degree polynomial, Y = aX + b, where Y was the peak area of PG/IS, a and b were constants, and X was the concentration (ng/mL) of the corresponding PG.

The results exhibited good linearity (r > 0.9930) of metabolites in linear ranges. Table 2 shows the linear calibration curve with correlation coefficients (r), linear ranges, the lower limit of detection (LOD), and the lower limit of quantitation (LOQ) of nine PGs both in the cell supernatant and rat serum. The signal of each analyte achieved a signal-to-noise ratio of 3 as its LOD and 10 as LOQ. The nanogram-graded LODs and LOQs indicated the machine’s high sensitivity.

**TABLE 2:** Linearity, LODs, and LOQs of the nine prostaglandins (PGs).

Analyte	Sample	Linearity			LOD	LOQ
		Calibration curve	*r*	Rang (ng/mL)	(ng/mL)	(ng/mL)
PGF_2β_	Supernatant	y = 1.98x-0.0400	0.9982	8.35–533	2.78	8.35
	Serum	y = 12.5x+0.131	0.9960	16.7–1.07 × 10^3^	5.57	16.7
PGE_2_	Supernatant	y = 21.2x-0.259	0.9980	2.17–555	0.723	2.17
	Serum	y = 110.4x+3.21	0.9975	0.867–1.10 × 10^3^	0.289	0.865
PGE_1_	Supernatant	y = 49.1x-0.500	0.9985	4.30–275	1.43	4.30
	Serum	y = 243.8x+5.23	0.9955	4.30–549	1.43	4.30
PGD_1_	Supernatant	y = 4.09x-0.0314	0.9965	3.13–200	1.05	3.13
	Serum	y = 76.0x+1.68	0.9960	3.15–200	1.05	3.15
PGD_2_	Supernatant	y = 4.03x-0.232	0.9975	8.75–560	2.92	8.75
	Serum	y = 646.23x+0.457	0.9936	0.875–1.12 × 10^3^	0.292	0.875
PGA_2_	Supernatant	y = 48.5x-0.00965	0.9980	0.781–50.0	0.260	0.781
	Serum	y = 6,051.63x+0.565	0.9908	0.0781–100	0.0391	0.0781
PGJ_2_	Supernatant	y = 34.8x-0.315	0.9985	1.17–150	0.390	1.17
	Serum	y = 136.94x+14.5	0.9970	2.30–150	0.767	2.30
PGB_2_	Supernatant	y = 10.5x-0.0443	0.9980	1.55–200	0.517	1.55
	Serum	y = 62.8x+1.50	0.9970	3.15–200	1.05	3.15
PGA_1_	Supernatant	y = 4.86x-0.0330	0.9980	2.88–185	0.960	2.88
	Serum	y = 24.7x+0.574	0.9985	5.75–369	1.92	5.75

#### 3.3.3 Precision and recovery

Precision and recovery investigations were performed to test the same sample at high, medium, and low concentrations. Their corresponding relative standard deviations (RSDs %) of intraday precision and interday precision were less than 15% (Table 3), and the recovery range was between 85% and 105% with RSDs of less than 15% (Table 3). These results indicated that the instrument met the criteria for biological sample analysis.

**TABLE 3:** Intraday precision, interday precision, and recovery of the nine prostaglandins (PGs).

Analyte	Sample	Theoretical conc. (ng/mL)	Intraday			Interday			Recovery	
			Conc. ± SD (ng/mL)	RSD (%)	RE (%)	Conc. ± SD (ng/mL)	RSD (%)	RE (%)	Mean ± SD (%)	RSD (%)
PGF_2β_	Supernatant	16.7	16.6 ± 0.100	0.535	−0.322	15.9 ± 1.62	10.2	−4.62	100 ± 3.30	3.30
		66.8	66.8 ± 1.00	1.47	0.0125	66.9 ± 0.0700	0.111	0.141	100 ± 1.36	1.36
		427	434 ± 4.60	1.06	1.48	429 ± 3.92	0.914	0.495	99.7 ± 1.10	1.11
	Serum	33.4	33.3 ± 0.260	0.795	−0.299	33.3 ± 0.450	1.35	−0.333	98.3 ± 1.25	1.27
		134	134 ± 0.300	0.225	0	135 ± 1.45	1.07	1.24	102 ± 1.09	1.07
		854	854 ± 2.20	0.257	0.00781	810 ± 71.2	8.79	−5.13	99.4 ± 0.773	0.778
PGE_2_	Supernatant	17.3	17.5 ± 0.300	1.84	1.09	17.5 ± 0.250	1.46	1.05	99.6 ± 2.33	2.34
		69.4	70.0 ± 0.400	0.615	0.836	69.6 ± 0.290	0.420	0.349	100 ± 0.144	0.144
		444	445 ± 3.10	0.702	0.286	444 ± 1.05	0.237	0.0592	100 ± 0.343	0.342
	Serum	17.3	17.5 ± 0.150	0.875	0.963	17.6 ± 0.200	1.15	1.54	100 ± 0.0384	0.0384
		139	138 ± 0.600	0.434	−0.360	138 ± 2.97	2.14	−0.328	102 ± 0.901	0.885
		888	883 ± 8.73	0.989	−0.604	895 ± 11.1	1.15	0.796	104 ± 0.373	0.358
PGE_1_	Supernatant	8.60	8.70 ± 0.200	2.22	1.74	8.70 ± 0.210	2.46	1.17	103 ± 2.76	2.67
		34.3	35.3 ± 1.40	3.99	2.97	34.5 ± 0.770	2.23	0.428	99.6 ± 1.02	1.03
		220	223 ± 5.90	2.66	1.36	220 ± 4.40	2.00	−0.033	97.7 ± 7.14	7.31
	Serum	8.60	8.83 ± 0.0600	0.654	2.71	8.75 ± 0.0700	0.806	1.78	88.4 ± 4.19	4.74
		68.6	68.7 ± 0.360	0.525	0.146	68.2 ± 0.650	0.956	−0.648	98.3 ± 0.421	0.428
		440	448 ± 1.46	0.327	1.81	446 ± 1.86	0.417	1.34	101 ± 0.656	0.650
PGD_1_	Supernatant	6.30	6.80 ± 0.400	5.29	7.63	6.65 ± 0.190	2.80	5.56	102 ± 3.24	3.17
		25.0	25.4 ± 0.900	3.48	1.50	25.4 ± 0.130	0.527	1.39	101 ± 1.46	1.44
		160	161 ± 2.40	1.50	0.466	164 ± 3.73	2.27	2.63	105 ± 0.998	0.949
	Serum	6.30	6.60 ± 0.170	2.62	4.76	6.48 ± 0.130	2.08	2.82	100 ± 1.58	1.58
		50.0	50.9 ± 1.37	2.68	1.73	50.8 ± 0.0900	0.174	1.60	102 ± 1.70	1.67
		180	182 ± 0.990	0.541	1.31	183 ± 0.750	0.408	1.70	105 ± 5.49	5.22
PGD_2_	Supernatant	17.5	17.3 ± 0.200	1.29	−1.42	17.4 ± 0.160	0.924	−0.401	99.8 ± 3.36	3.36
		70.0	70.9 ± 2.20	3.04	1.24	70.7 ± 0.340	0.476	0.940	100 ± 0.940	0.937
		448	440 ± 8.32	1.89	−1.69	444 ± 3.15	0.924	−0.935	99.5 ± 0.852	0.857
	Serum	1.75	1.72 ± 0.0219	1.27	−1.49	1.70 ± 0.0668	3.94	−2.95	99.6 ± 0.627	0.630
		140	141 ± 0.460	0.325	0.571	142 ± 1.92	1.36	1.13	103 ± 1.15	1.12
		896	898 ± 8.41	0.937	0.186	894 ± 5.42	0.607	−0.224	99.1 ± 0.500	0.504
PGA_2_	Supernatant	1.60	1.80 ± 0.100	5.01	9.76	1.78 ± 0.0900	4.86	11.0	103 ± 3.10	3.02
		6.30	6.80 ± 0.100	1.94	7.86	6.51 ± 0.25	3.85	3.27	101 ± 4.54	4.50
		40.0	41.2 ± 1.00	2.42	2.98	41.7 ± 1.28	3.06	4.14	102 ± 0.543	0.534
	Serum	1.90	1.93 ± 0.0360	1.87	1.37	1.91 ± 0.0396	2.07	0.386	101 ± 1.02	1.01
		12.6	12.5 ± 0.0600	0.463	−1.06	12.4 ± 0.170	1.35	−1.24	100 ± 2.86	2.86
		80.0	81.0 ± 0.460	0.566	1.25	83.4 ± 2.85	3.42	4.20	108 ± 3.09	2.85
PGJ_2_	Supernatant	2.30	2.60 ± 0.100	3.69	14.6	2.59 ± 0.140	5.53	12.5	105 ± 10.6	10.0
		9.40	9.30 ± 0.200	1.80	−0.976	9.40 ± 0.140	1.51	0.0433	99.3 ± 1.95	1.97
		120	119 ± 3.90	3.25	−0.688	119 ± 0.750	0.624	−0.477	99.0 ± 2.09	2.11
	Serum	4.60	4.53 ± 0.120	2.55	−1.45	4.59 ± 0.0500	1.11	−0.242	101 ± 3.32	3.30
		18.8	18.8 ± 0.0600	0.307	0.177	18.8 ± 0.160	0.839	−0.118	98.9 ± 1.41	1.42
		120	125 ± 0.550	0.442	3.89	123 ± 2.69	2.18	2.67	104 ± 2.14	2.06
PGB_2_	Supernatant	3.10	3.30 ± 0.200	4.54	7.53	3.44 ± 0.100	2.93	11.0	111 ± 2.97	2.66
		12.3	12.5 ± 0.400	2.85	1.44	12.8 ± 0.400	3.16	4.23	103 ± 3.04	2.94
		160	163 ± 2.20	1.37	1.65	160 ± 2.15	1.34	0.124	99.6 ± 1.13	1.14
	Serum	6.30	6.37 ± 0.250	3.95	1.06	6.24 ± 0.120	1.87	−0.882	98.9 ± 2.42	2.45
		25.0	25.1 ± 0.360	1.44	0.400	25.3 ± 0.230	0.925	1.29	101 ± 1.06	1.05
		160	163 ± 4.20	2.58	1.73	166 ± 2.52	1.52	3.49	104 ± 0.556	0.534
PGA_1_	Supernatant	5.80	6.40 ± 0.400	6.48	10.5	6.04 ± 0.330	5.43	4.20	110 ± 2.95	2.87
		23.1	23.8 ± 0.200	0.853	2.96	23.5 ± 0.250	1.06	1.83	101 ± 1.89	1.88
		148	148 ± 1.10	0.774	0.471	148 ± 0.330	0.223	0.214	100 ± 0.476	0.476
	Serum	11.5	11.5 ± 0.0600	0.501	0.290	11.6 ± 0.120	0.994	1.06	101 ± 2.01	1.98
		46.1	46.5 ± 0.250	0.541	0.940	46.3 ± 0.190	0.401	0.506	100 ± 1.44	1.44
		295	295 ± 2.31	0.782	−0.0340	295 ± 2.97	1.01	−0.0410	100 ± 0.928	0.928

#### 3.3.4 Stability

The results indicated that biological samples were stable enough for quantitative determination at room temperature, at −20°C, or freeze–thaw cycles. Table 4 shows the RSD results of the nine PGs’ stability, which were less than 15% in the cell supernatant and rat serum samples with high, medium, and low concentrations, respectively.

**TABLE 4:** Stability of the nine prostaglandins (PGs).

Analyte	Sample	Theoretical Conc. (ng/mL)	Room temperature for 6 h		−20 °C for 30 days		Freeze-thaw for triplicate	
			Conc. ± SD (ng/mL)	RSD (%)	Conc. ± SD (ng/mL)	RSD (%)	Conc. ± SD (ng/mL)	RSD (%)
PGF_2β_	Supernatant	16.7	16.7 ± 0.400	2.16	16.8 ± 0.300	1.78	17.1 ± 0.230	1.35
		66.8	66.7 ± 0.200	0.346	67.3 ± 0.200	0.261	67.6 ± 0.350	0.525
		427	429 ± 2.80	0.653	432 ± 7.40	1.71	425 ± 1.39	0.326
	Serum	33.4	33.5 ± 0.230	0.690	33.2 ± 0.210	0.628	33.8 ± 0.450	1.33
		134	137 ± 3.15	2.30	135 ± 0.930	0.689	134 ± 0.700	0.522
		854	849 ± 4.16	0.489	851 ± 2.15	0.253	726 ± 3.39	0.467
PGE_2_	Supernatant	17.3	14.2 ± 0.400	2.47	17.3 ± 0.300	1.49	17.5 ± 0.200	1.15
		69.4	56.1 ± 0.600	1.07	69.7 ± 0.900	1.29	69.8 ± 0.430	0.616
		444	363 ± 2.70	0.733	445 ± 1.70	0.373	445 ± 0.950	0.214
	Serum	17.3	17.3 ± 0.0200	0.110	17.6 ± 0.310	1.74	17.7 ± 0.170	0.979
		139	142 ± 2.21	1.56	137 ± 0.800	0.585	138 ± 0.320	0.232
		888	921 ± 4.43	0.481	889 ± 6.12	0.688	892 ± 4.45	0.500
PGE_1_	Supernatant	8.60	8.60 ± 0.300	3.08	8.80 ± 0.100	0.680	9.01 ± 0.380	4.25
		34.3	33.5 ± 1.30	3.78	34.2 ± 0.800	2.32	34.2 ± 0.230	0.679
		220	218 ± 6.20	2.85	228 ± 1.10	0.485	223 ± 0.580	0.258
	Serum	8.60	7.57 ± 0.320	4.25	8.80 ± 0.200	2.27	8.60 ± 0.260	3.08
		68.6	78.9 ± 0.920	1.16	68.7 ± 0.510	0.747	67.9 ± 1.15	1.70
		440	445 ± 2.10	0.470	443 ± 1.91	0.432	440 ± 0.0700	0.0161
PGD_1_	Supernatant	6.30	6.90 ± 0.200	2.20	6.80 ± 0.300	4.69	6.19 ± 0.110	1.77
		25.0	25.3 ± 0.300	0.993	25.3 ± 0.400	1.63	25.2 ± 0.320	1.26
		160	166 ± 1.50	0.893	166 ± 4.20	2.54	165 ± 3.37	2.04
	Serum	6.30	6.22 ± 0.100	1.53	6.47 ± 0.210	3.22	6.37 ± 0.380	5.95
		50.0	53.4 ± 0.810	1.51	50.8 ± 1.00	1.97	51.8 ± 0.380	0.731
		180	176 ± 3.80	2.16	181 ± 0.760	0.422	182 ± 0.420	0.232
PGD_2_	Supernatant	17.5	17.6 ± 0.200	0.87	18.1 ± 0.300	1.55	17.4 ± 0.220	1.29
		70.0	69.7 ± 0.300	0.461	70.5 ± 0.200	0.301	69.1 ± 0.570	0.832
		448	447 ± 2.50	0.560	446 ± 1.50	0.340	446 ± 2.88	0.644
	Serum	1.75	1.73 ± 0.0244	1.41	1.76 ± 0.0214	1.21	1.74 ± 0.0238	1.19
		140	141 ± 1.69	1.20	141 ± 0.200	0.142	139 ± 0.530	0.379
		896	895 ± 11.6	1.30	898 ± 7.00	0.779	895 ± 4.88	0.545
PGA_2_	Supernatant	1.60	1.50 ± 0.100	6.67	1.80 ± 0.200	11.3	1.81 ± 0.0400	2.24
		6.30	6.50 ± 0.200	2.36	6.60 ± 0.400	5.92	6.39 ± 0.160	2.45
		40.0	41.3 ± 0.300	0.740	43.5 ± 2.10	4.86	42.4 ± 2.21	5.21
	Serum	1.90	1.93 ± 0.0788	4.09	1.92 ± 0.0351	1.83	1.93 ± 0.0232	1.20
		12.6	12.0 ± 0.120	0.960	12.4 ± 0.0600	0.464	12.4 ± 0.400	3.25
		80.0	85.3 ± 0.950	1.12	80.7 ± 0.600	0.747	83.1 ± 1.70	2.04
PGJ_2_	Supernatant	2.30	2.50 ± 0.300	10.6	2.60 ± 0.200	7.39	2.48 ± 0.150	5.86
		9.40	9.30 ± 0.200	2.15	9.40 ± 0.300	2.95	9.45 ± 0.200	2.13
		120	118 ± 0.600	0.542	124 ± 3.10	2.54	121 ± 3.03	2.50
	Serum	4.60	4.40 ± 0.170	3.94	4.57 ± 0.230	5.06	4.67 ± 0.150	3.27
		18.8	18.1 ± 0.100	0.552	18.9 ± 0.230	1.22	18.8 ± 0.210	1.11
		120	123 ± 2.34	1.90	123 ± 0.400	0.329	119 ± 0.420	0.358
PGB_2_	Supernatant	3.10	3.50 ± 0.200	6.81	3.40 ± 0.100	3.80	3.31 ± 0.210	6.44
		12.3	12.8 ± 0.500	3.85	13.0 ± 0.900	6.88	12.7 ± 0.190	1.52
		160	165 ± 3.53	2.14	172 ± 7.00	4.10	160 ± 1.29	0.806
	Serum	6.30	6.20 ± 0.170	2.79	6.40 ± 0.170	2.71	6.33 ± 0.0600	0.912
		25.0	25.5 ± 0.120	0.452	25.1 ± 0.250	1.00	25.2 ± 0.700	2.78
		160	162 ± 1.87	1.16	161 ± 0.950	0.587	165 ± 1.70	1.03
PGA_1_	Supernatant	5.80	5.70 ± 0.300	4.64	5.70 ± 0.300	5.02	5.87 ± 0.260	4.38
		23.1	23.5 ± 0.200	0.737	23.0 ± 0.300	1.41	23.0 ± 0.230	1.00
		148	147 ± 1.90	1.30	148 ± 1.60	1.09	148 ± 0.530	0.358
	Serum	11.5	11.6 ± 0.190	1.67	11.3 ± 0.100	0.885	11.5 ± 0.210	1.82
		46.1	46.3 ± 0.550	1.19	46.1 ± 0.320	0.697	46.5 ± 0.290	0.621
		295	295 ± 1.47	0.500	297 ± 0.590	0.197	293 ± 0.280	0.0964

### 3.4 Contents of PGs in samples

**FIGURE 4:**
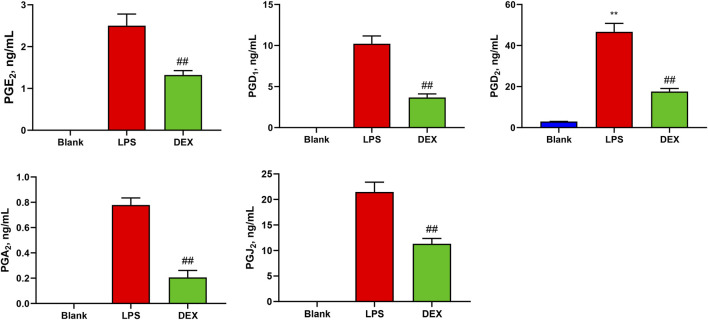
Levels of prostaglandins (PGs) in the samples of cell supernatant. The levels of PGE_2_, PGD_1_, PGD_2_, PGA_2_, and PGJ_2_ in RAW264.7 cell supernatants were increased after LPS stimulation, while these levels were significantly decreased after the intervention of the positive drug (dexamethasone). Paired t-tests were used for statistical analyses. ##*P* < 0.01, vs. LPS group, *n* = 6.

In this study, a novel UHPLC-QQQ-MS/MS method was well-established for the simultaneous quantification of nine PGs in cell supernatant samples and rat serum samples. Figure 4 shows that the levels of PGE_2_ (2.50 ± 0.253 ng/mL), PGD_1_ (10.2 ± 0.869 ng/mL), PGD_2_ (46.7 ± 3.73 ng/mL), PGA_2_ (0.778 ± 0.0512 ng/mL), and PGJ_2_ (21.5 ± 1.74 ng/mL) in RAW264.7 cell supernatants were increased after LPS stimulation. At the same time, these levels were significantly decreased by the treatment with an anti-inflammatory drug (*p* < 0.01). Meanwhile, the PGF_2β_, PGE_1_, PGB_2_, and PGA_1_ levels in RAW 264.7 cell supernatants were lower than LOQs.

**FIGURE 5:**
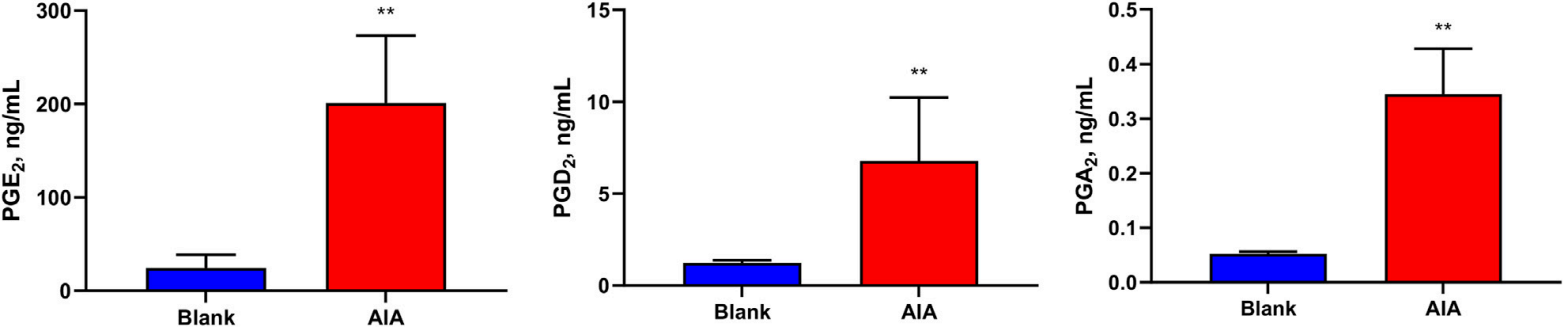
Levels of prostaglandins (PGs) in the samples of rat serum. The levels of PGE_2_, PGA_2_, and PGD_2_ were significantly increased in the serum of AIA rats. Paired t-tests were used for statistical analyses. **P < 0.01, vs. blank group, *n* = 6.

In addition, Figure 5 shows that the levels of PGE_2_ (201 ± 66.0 ng/mL), and its metabolite PGA_2_ (0.345 ± 0.0758 ng/mL), and PGD_2_ (6.78 ± 3.16 ng/mL) were significantly increased in the serum of AIA rats (*p* < 0.01). The levels of other PGs were less than those of LOQs in the rat serum.

## 4 Discussion

In this study, we established an LC-MS method capable of simultaneously determining nine PGs with nanogram-graded sensitivity and successfully applied this method to the classical *in vivo* and *in vitro* inflammation models. The advantage of this method lies in its ability to rapidly determine the contents of nine PGs within 8 min simultaneously, which exhibits higher detection efficiency than conventional ELISA and WB methods that take hours.

As all know, RAW264.7 macrophages are commonly utilized as an inflammatory model *in vitro*. PG activation plays a vital role in the occurrence and development of inflammation and immune response (Yao and Narumiya, 2019; Maseda et al., 2019). cPLA2 expression is increased by LPS in RAW cells, producing AA, which is then converted into PGs through the COXs metabolic pathway (Pan et al., 2022; Zhang et al., 2023). The results of the LC-MS method established in this study showed that PGs were lowly expressed in normal RAW264.7 cells. The PGE_2_, PGD_2_, PGA_2_, and PGJ_2_ expression was significantly elevated in the cells after LPS induction, which induced inflammatory responses. In contrast, the positive drug (DEX) reduced the levels of these PGs and exerted anti-inflammatory effects. The above results mainly agree with the trend of results reported by conventional ELISA kit assays (Zhou et al., 2021; Baris et al., 2024) and previous studies by UPLC-MS/MS (Wang D. et al., 2023). Interestingly, anti-inflammatory PGs, except PGD_1_ *in vitro,* were not detected in both *in vivo* and *in vitro* experiments, while pro-inflammatory PGs were decreased in the positive drug group. It is speculated to be related to the anti-inflammatory mechanism of DEX. This is because DEX directly inhibits the activity of upstream COXs and reduces the production of pro-inflammatory PGs rather than interfering with the level of downstream anti-inflammatory PGs (Park et al., 2023).

AIA is a classic model of rheumatoid arthritis (RA), which is characterized by inflammatory cell infiltration and joint bone damage. The AIA model simulates the pathological changes of arthritis mediated by exogenous factors. Due to the structural similarity between a protein molecule of *Mycobacterium tuberculosis* and a glycoprotein molecule on the synovial membrane of joints, it can be recognized by T-cells, thereby inducing an immune response against joints (Nakamachi et al., 2016; Hegen et al., 2008). An increasing amount of evidence indicates that the inflammation in RA is associated with the release of PGs. PGD_2_ (Malik et al., 2023), PGE_2_ (Ballerini et al., 2022), and PGB_2_ (Carnovale et al., 2022) play a role in promoting inflammatory cell infiltration, synovial hyperplasia, and angiogenesis by regulating the differentiation and maturation of immune cells and the production of cytokines, thereby exacerbating the inflammatory response within the joints. Second, PGE_2_ (Pujol and Loyau, 1987) and PGA_2_ (Ohmura et al., 2017) can also be involved in the degradation of articular cartilage and bone resorption processes, which consequently lead to bone destruction in the joints. Furthermore, PGF_2β_ (Quinteiro et al., 2012), PGJ_2_ (Calder, 2020), and PGE_2_ (Globig et al., 2025) serve as significant mediators in the regulation of joint pain. Conversely, PGE_1_ (Braune et al., 2020), PGD_1_ (Maher et al., 2015), and PGA_1_ (Lee et al., 2021) reduces inflammation by inhibiting platelet aggregation, neutrophil activation, and the NF-κB pathway.

This study revealed that the levels of PGD_2_ and PGE_2_ and its metabolites PGA_2_ in the serum of AIA rat models were significantly elevated. The increases in the PGs levels contributed to the aggravation of vascular dilation and edema in the model group (Kondeti et al., 2016), which manifested as the paw swelling. This demonstrated that AIA successfully induced arthritis in rats. Moreover, it indicates that the LC-MS method established in this study can effectively detect inflammatory indicators in AIA-induced arthritis rats.

Moreover, the expression levels of different PGs can also reflect the different stages of inflammation. For example, AA is converted into different PGs, from PGE_2_ in the acute phase to PGD_2_ in the regression phase (Liu et al., 2023; Montrose et al., 2015). The upregulation of PGE_2_ accelerated the development of arthritis (Lee et al., 2020), while an increased PGD_2_ indicates that the inflammation has entered a resolution phase (Kapoor et al., 2007). The phenomenon of elevated PGE_2_ and PGD_2_ simultaneously appeared in the model rats, which requires kinetic analysis of PGs in the AIA model to be explored.

Finally, due to the cost and time limitation, this study has some shortcomings, one of which was the failure to use the ELISA method for parallel detection of samples. The results of comparison between the two methods can further support the conclusion of this study. We also did not measure the contents of PGG_2_, PGH_2_, PGF_2α_, and PGC_2_ in the AA pathway due to the lack of the corresponding reference compounds. Compared with the traditional ELISA, the UHPLC-QQQ-MS/MS method has certain acknowledged limitations, such as in terms of sensitivity. Detecting low-abundance PGs also poses a challenge for the LC-MS method. As a result, PGs in some groups in this experiment could not be detected by this method.

## 5 Conclusion

In this study, a novel LC-MS method for the simultaneous determination of nine PGs was successfully established, which can quickly and sensitively achieve the concentration of PGs in biological samples within a wide detection range, high specificity and accuracy, and low sample demand. The method has been verified by *in vitro* and *in vivo* inflammatory model samples and can provide a new tool for detecting PGs in the AA metabolic pathway.

## Data Availability

The original contributions presented in the study are included in the article/Supplementary Material; further inquiries can be directed to the corresponding author.
