# Supplementary material for: Determination of nine prostaglandins in the arachidonic acid metabolic pathway with UHPLC-QQQ-MS/MS and application to in vitro and in vivo inflammation models
Source: Front Pharmacol. 2025 May 30;16:1595059. doi: 10.3389/fphar.2025.1595059 (PMC12162277; doi:10.3389/fphar.2025.1595059)
Supplement: Supplementary file 1 [file DataSheet1.docx]

Supplementary Material

## Supplementary Figures


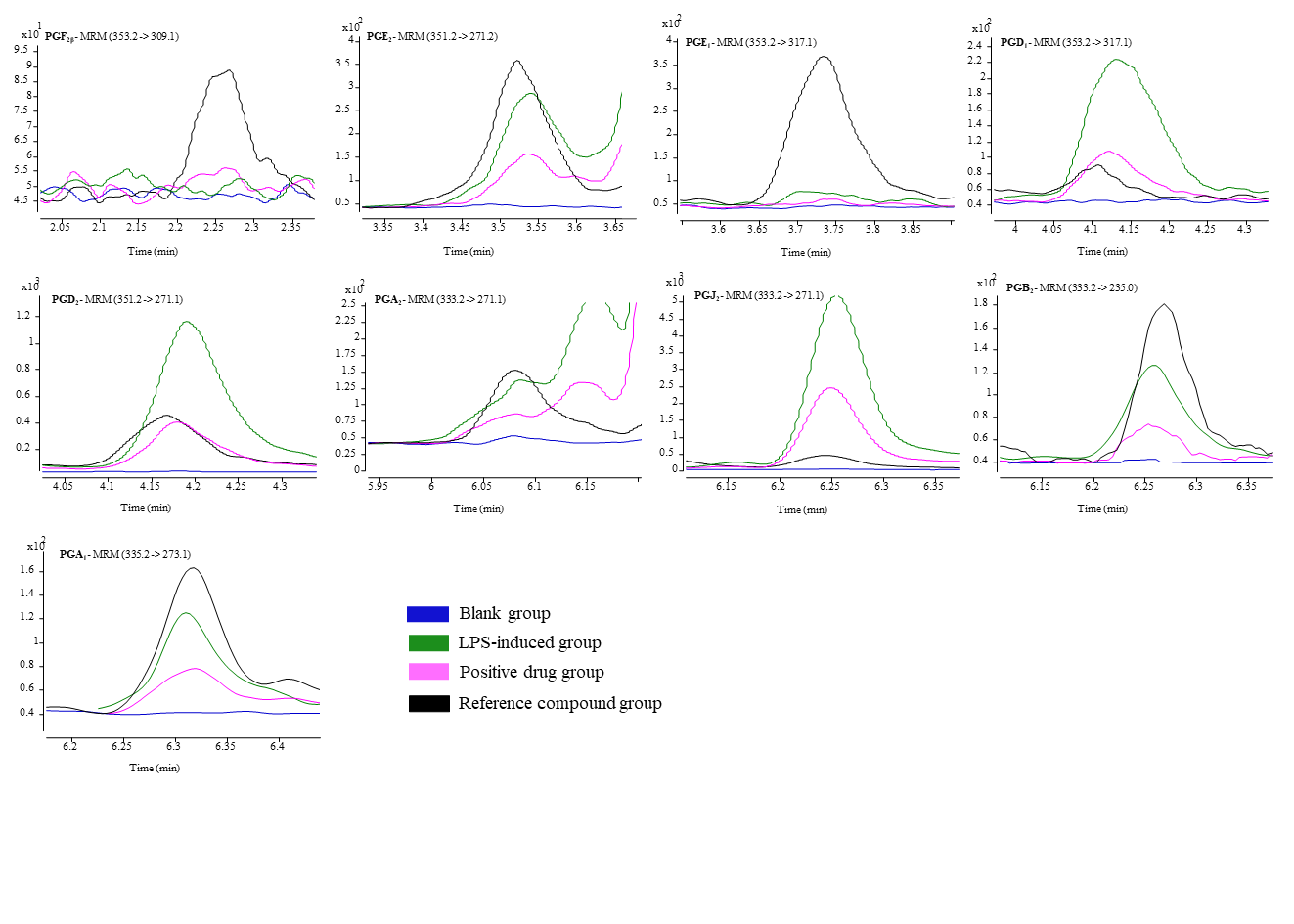


**Fig. S1.** The representative extract ion chromatograms (EICs) of the reference compounds and samples of cell supernatant.


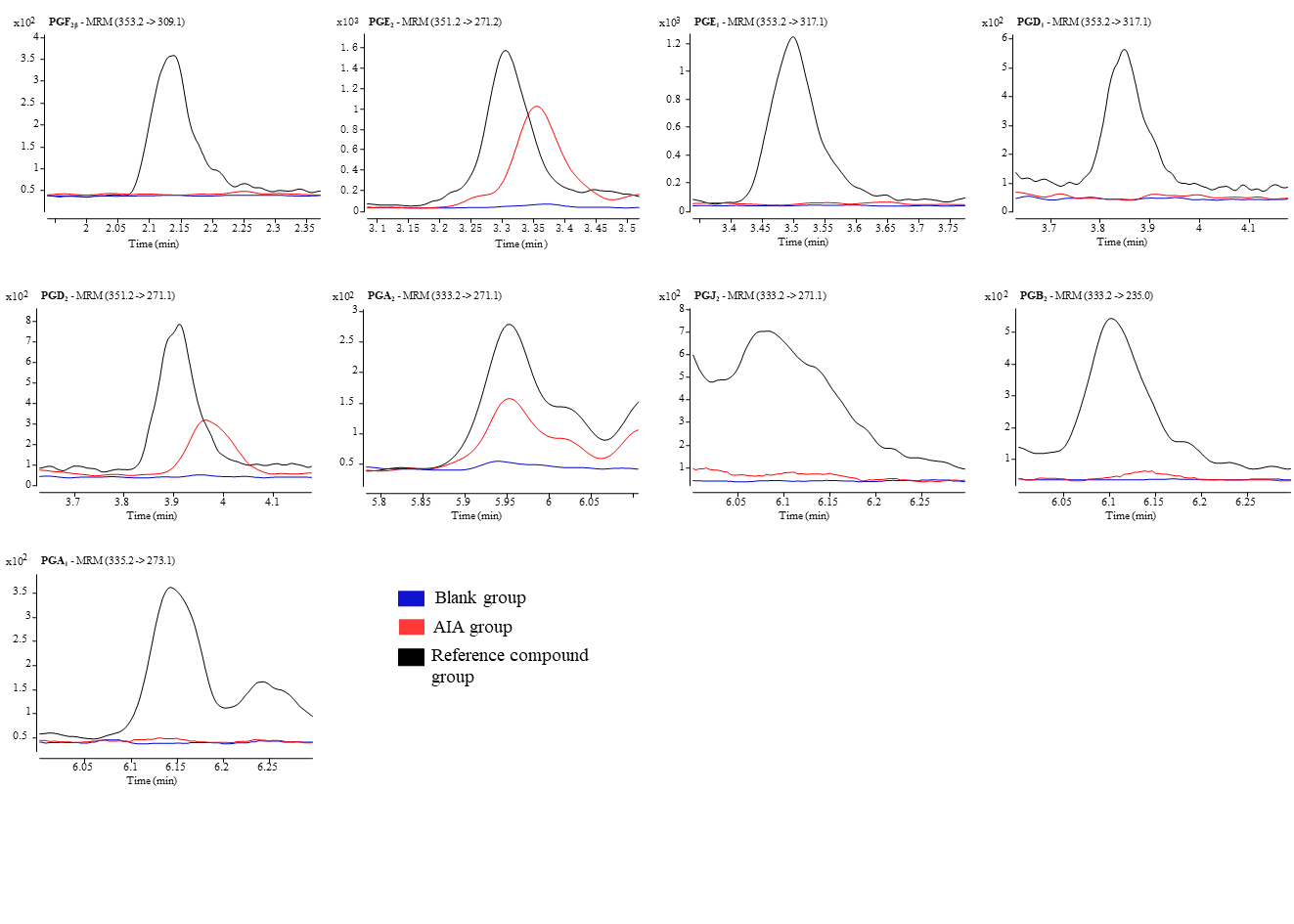


**Fig. S2.** The representative extract ion chromatograms (EICs) of the reference compounds and samples of rat serum.
